# Prevalence of Depression in Patients and Survivors of Breast Cancer: A Systematic Review

**DOI:** 10.7759/cureus.63816

**Published:** 2024-07-04

**Authors:** Farees Ahmad Khan, Jason A Castillo, Kavya Sai Satya Amaravadi, Poornachandra Nalisetty, Nandini Vadlamani, Sabina Ibrahimli, Pousette Hamid

**Affiliations:** 1 Medicine, California Institute of Behavioral Neurosciences & Psychology, Fairfield, USA; 2 Internal Medicine, California Institute of Behavioral Neurosciences & Psychology, Fairfield, USA; 3 Surgery, California Institute of Behavioral Neurosciences & Psychology, Fairfield, USA; 4 Family Medicine, California Institute of Behavioral Neurosciences & Psychology, Fairfield, USA; 5 Neurology, California Institute of Behavioral Neurosciences & Psychology, Fairfield, USA

**Keywords:** survivors, patients, breast carcinoma, breast malignancy, breast cancer, mood disorder, depression

## Abstract

Depression is an illness prevalent worldwide and much more common in certain groups of people. Individuals suffering from breast cancer as well as the survivors of breast cancer are at an increased risk of developing depression. We conducted this systematic review using articles from different countries of the world to get an estimate of the prevalence of depression in this specified population. For this, we collected about 262 articles from Google Scholar, PubMed, and ScienceDirect, and after strict scrutiny, 13 articles were used to extract our data. From our collected data, we were able to get an estimate of depression prevalence rates among breast cancer patients and survivors globally and identify different factors that affected these rates. More cohort studies must be done so that more precise information about the causes, preventions, and therapies of depression specifically in breast cancer patients and survivors may be gathered.

## Introduction and background

“My diagnosis flung me into despair until it hit me: I’m alive.” - Dana Dinerman [1]. Depression is a type of mood disorder that according to the World Health Organization (WHO) is said to affect about 5% of the adult population worldwide [2]. According to DSM-5-TR diagnostic criteria, major depressive disorder is diagnosed when a person experiences for a duration of two weeks five or more symptoms of depression [3]. These symptoms must include depressed mood or loss of interest or pleasure and must exclude symptoms attributed to another medical condition. The other symptoms of depression mentioned in DSM-5-TR include decreased or increased weight with change of appetite nearly every day, fatigue, insomnia or hypersomnia, feelings of worthlessness or guilt, decreased concentration, psychomotor agitation or retardation, thoughts of death or suicide. Additional requirements of the DSM-5-TR are as follows: 1. The symptoms cause significant distress or social, functional, or emotional impairment. 2. The symptoms are not due to any substance use or any other medical conditions. 3. There is no history of mania or hypomania. 4. The episodes are not due to schizoaffective disorder, schizophrenia, schizophreniform disorder, delusional disorder, or any specific and unspecific schizophrenia spectrum and other psychotic disorders [3]. 

Depression can occur for a variety of reasons, including social, psychological, and biological aspects [2]. Depression is more likely to occur in people who have undergone catastrophic life events, such as unemployment, trauma, or bereavement. Compared to healthy people, those who have health conditions including cancer, diabetes, cardiovascular disease, or respiratory disorders are more prone to experience depression [2]. Breast cancer is a disease in which cells of the breast start dividing uncontrollably due to some error in the normal mitotic division. There are different types of breast cancer identified; the type depends on the type of cell that becomes faulty [4]. Cancer can originate from the cells of the lobules, ducts, or connective tissue of the breast. The cancerous cells can spread from their area of origin to other areas of the body through blood or the lymphatic system; if this happens the cancer is said to be malignant. WHO reports 685000 deaths worldwide and 2.3 million new breast cancer diagnoses in women occurred in 2020 [5]. According to various pieces of literature, people who are either breast cancer patients or survivors experience varying levels of depression [6-16]. Depression can adversely affect the patients by affecting their will to fight the disease as well as negatively impacting the survivors' quality of life after they have won the battle against breast cancer. Numerous therapeutic modalities have been created to both treat and prevent depression in these patients and survivors such as individual counseling, antidepressant medication, or support groups [17]. There is still some uncertainty on whether there is a significant prevalence of depression among people who have or have had breast cancer. To establish a clear connection between depression and breast cancer, we intend to conduct a systematic review of the literature that is currently available internationally. This would assist future physicians to watch out for depression among breast cancer patients and long-term survivors.

## Review

Methodology

We followed the Preferred Reporting for Systematic Reviews and Meta-Analyses (PRISMA) standards for conducting our systematic review [18].

Database

We began our research on the 30th of June 2023 using the online libraries as our databases. During our search for data, we collected articles from PubMed, Google Scholar, and ScienceDirect.

Search Strategy

We included articles related to depression and breast cancer. For our advanced search, we used keywords from the building block technique, which included depression, mood disorders, bipolar disease, breast cancer, breast malignancy, and breast carcinoma. The keywords were combined using the Boolean operators "AND" and "OR." The results of our search are shown in Table 1.

**Table 1:** Initial search results data

Database	Keywords	Initial results	Results after filters applied
PubMed	[Depression OR mood disorder OR bipolar disease]	672128	
	[Breast cancer OR breast malignancy OR breast carcinoma]	504325	
	[[Depression OR mood disorder OR bipolar disease]] AND [[Breast cancer OR breast malignancy OR breast carcinoma]]	6319	128
Google Scholar	“Depression” AND “Breast Cancer”	885000	17800
ScienceDirect	“Depression” AND “Breast Cancer”	34640	1979

Inclusion Criteria

The articles included in this study were peer-reviewed and of relevance to our systematic review topic. Data items collected from the selected articles included the prevalence rates of depression among breast cancer survivors and patients and contributing factors to the prevalence of depression among them. They were majorly observational studies done from the year 2020 to 2023 in the English language.

Exclusion Criteria

To maintain the integrity and focus of our review, we implemented strict exclusion criteria. Articles falling under the following categories were eliminated from consideration: 1. Grey literature, including unpublished works, dissertations, and conference abstracts. 2. Animal studies. 3. Case reports, case series, and randomized clinical trials. 4. Publications older than five years from the start of our research in June 2023. These criteria were established to ensure the selection of good quality and relevant studies aligning with the objectives of our systematic review.

Quality Assessment Tools

We used the New Castle-Ottawa scale (NOS) for the observational studies included in our systematic review to assess their quality and excluded studies that scored low. The NOS evaluates studies based on the selection of study groups, comparability of groups, and ascertainment of exposure or outcome.

Data Extraction and Collection

Data extraction was performed by two independent reviewers for the reduction of bias and error. Any discrepancies between the reviewers have been dealt with by discussion, consensus, and consultancy with a third reviewer. Extracted data included the characteristics of the studies, participant demographics, prevalence rates of depression, and factors associated with depression.

After checking the quality, data were retrieved from the final articles. The data were then entered into a standardized form for data extraction and managed using Microsoft Excel.

Results

After searching the databases, a total of 262 articles were collected, which included 33 articles from Google Scholar, 128 articles from PubMed, and 101 articles from ScienceDirect. All these articles were gathered into a table on Microsoft Excel in which eight duplicates were found and subsequently removed. The articles were then screened by looking at their titles (172 articles were removed) and then their abstracts (49 articles were removed) for relevance to the topic of our systematic review. After reviewing the full articles, six articles were removed because only their abstracts without full articles were available for free viewing, and a further 16 articles were removed because they did not have the relevant information for our topic.

**Figure 1:**
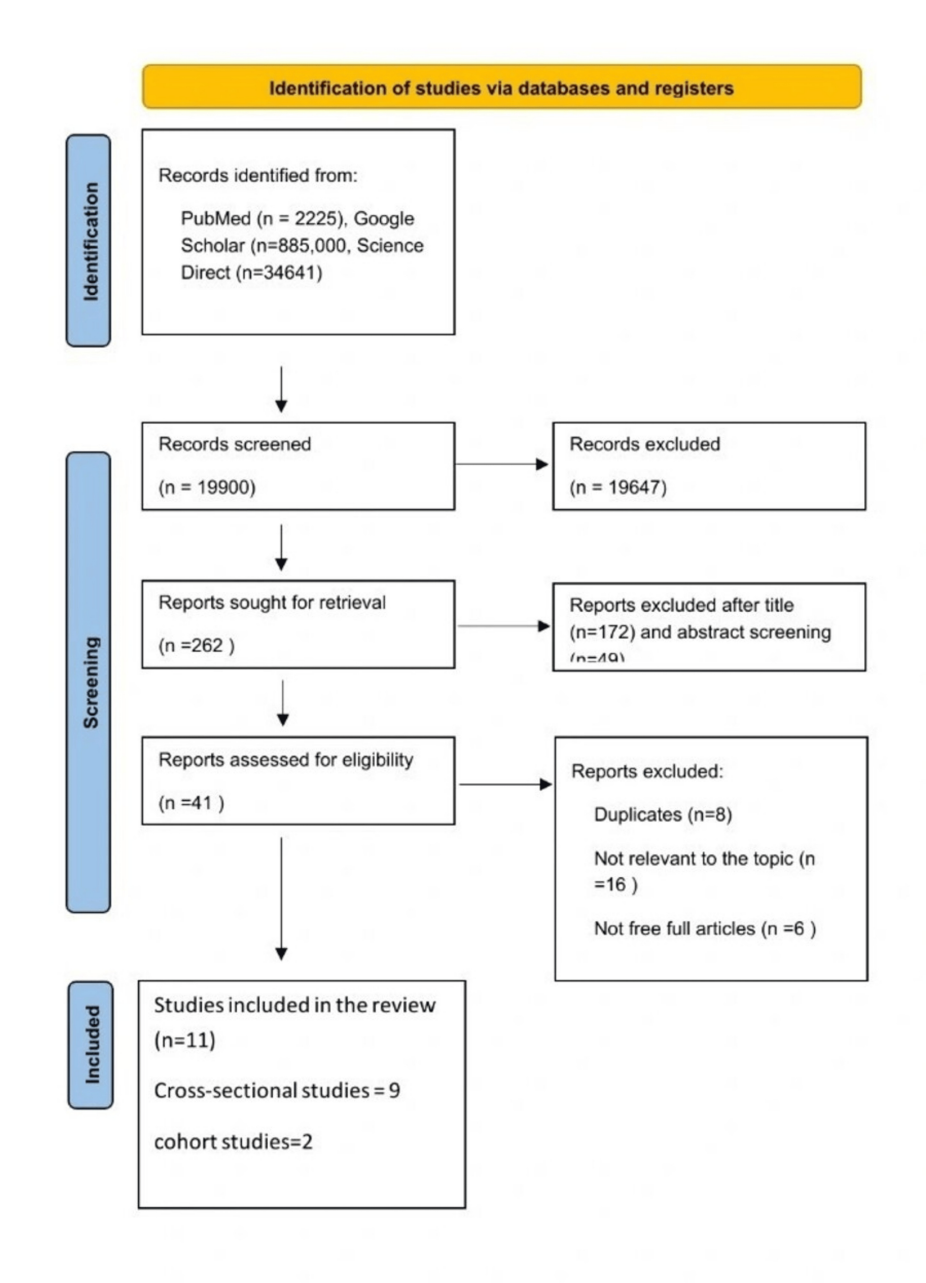
PRISMA flow diagram PRISMA, Preferred Reporting Items for Systematic Reviews and Meta-Analyses

For the assessment of the methodological quality of the articles, the Newcastle-Ottawa scale was used. This tool assessed the quality of the study on the basis of factors such as the selection of study groups, the comparability of groups, and the ascertainment of either the exposure or outcome of interest. Following this extensive assessment, 11 articles were found to have good methodological quality with relevant data to our review. This is depicted in Figure 1. 

Baseline Characteristics of Included Studies

The final number of articles included is 11 of which nine are observational cross-sectional studies and two are observational prospective cohort studies. The studies are from the years 2020 to 2023. Seven of the studies involve breast cancer survivors while the remaining four are focused on breast cancer patients. In all of the studies, patients with primary breast cancer are included and secondary breast cancer patients are excluded. Among these studies, in one the patients with breast cancers I-IV were included and in three studies the stages of breast cancer in patients were not mentioned. There is a total of 10781 people whose data are included in the studies, which are picked from various nations throughout the world. Table 2 outlines the characteristics of the 11 included studies.

**Table 2:** Table of characteristics of the studies included in this systematic review

Author	Journal and year of publication	Title	Country	Type of research	Number of subjects	Outcome
Sergio Álvarez-Pardo [6]	International Journal of Environmental Research and Public Health. 2023	Related factors with depression and anxiety in mastectomized women breast cancer survivors	Mexico	Cross- sectional study	198 survivors	10.6% had pathological depression
Jameel Soqia [7]	BMC Psychiatry. 2022	Depression, anxiety and related factors among Syrian breast cancer patients: a cross-sectional study	Syria	cross-sectional study	500 patients	About 35% had depression
Clara Breidenbach [8]	BMC Psychiatry. 2022	Prevalence and determinants of anxiety and depression in long-term breast cancer survivors	Germany	Cross-sectional study	164 survivors	34.9% of the 90.9% had depression after about 5-6 years
Annelieke A. Lemij [9]	European Journal of Cancer. 2023	Mental health outcomes in older breast cancer survivors: five-year follow-up from the CLIMB study	Netherlands	Cohort study	299 survivors	10.4% had depression after 3 months
Penelope Aggeli [10]	Asia-Pacific Journal of Oncology Nursing. 2021	Posttreatment anxiety, depression, sleep disorders, and associated factors in women who survive breast cancer	Greece	Cross-sectional study	170 survivors	18.2% reported depression
Barbara Muzzatti [11]	BMC Cancer. 2020	Quality of life and psychological distress during cancer: a prospective observational study involving young breast cancer female patients	Italy	Cross-sectional study	106 patients	During hospital stay 17.9% and 9.4% were respectively possible and probable cases of depression. 12 months later the possible and probable percentages were 8.5 and 6.6% for depression.
Murray Foster and Claire L. Niedzwiedz [12]	BMC Cancer. 2021	Associations between multimorbidity and depression among breast cancer survivors within the UK Biobank cohort: a cross-sectional study	England, Scotland, Wales	Cross-sectional study	8438 survivors	5.3% were having depression
Fernanda Elisa Ribeiro [13]	International Journal of Environmental Research and Public Health. 2023	Comparison of quality of life in breast cancer survivors with and without persistent depressive symptoms: a 12-month follow-up study	Brazil	Cross-sectional study	70 survivors	17.1% reported persistent symptoms of depression
Joana Perez-Tejada [14]	European Journal of Oncology Nursing. 2021	Anxiety and depression after breast cancer: The predictive role of monoamine levels	Spain	Cross-sectional study	107 survivors	27.8% had clinically significant depression symptoms
Dana Sadaqa [15]	BMC Cancer. 2022	Risk of developing depression among breast cancer patients in Palestine	Palestine	Cross-sectional study	223 patients	35.4% women suffered from moderate-severe depression symptoms
Catarina Lopes [16]	Current Oncology. 2022	Prevalence and persistence of anxiety and depression over five years since breast cancer diagnosis - The NEON-BC Prospective Study	Portugal	Cohort study	506 patients	The five-year period prevalence of depression was 25.5%

Discussion

Overview of the Included Studies

We included in our systematic review 11 observational studies with a total sample size of 10781 breast cancer patients and survivors. The studies collected were from different countries in North and South America, Europe, the Middle East, and Asia. The assessment tools used mostly in these studies to measure depression were the Hospital Anxiety and Depression Scale (HADS), the Center for Epidemiologic Studies-Depression Scale (CES-D), the Geriatric Depression Scale (GDS), Patient Health Questionnaire 9-item depression scale (PHQ-9), and the Generalized Anxiety Disorder-2 (GAD-2) scale. These instruments are specifically designed to differentiate grief from depression by focusing on pervasive affective, cognitive, and physical features of depression that have lasted for a long time. In contrast to grief, which may entail temporary sadness and mourning, these criteria guarantee the assessment of major depressive disorder characterized by persistent and impairing depressed mood.

Prevalence of Depression in Breast Cancer Patients and Survivors

**Figure 2:**
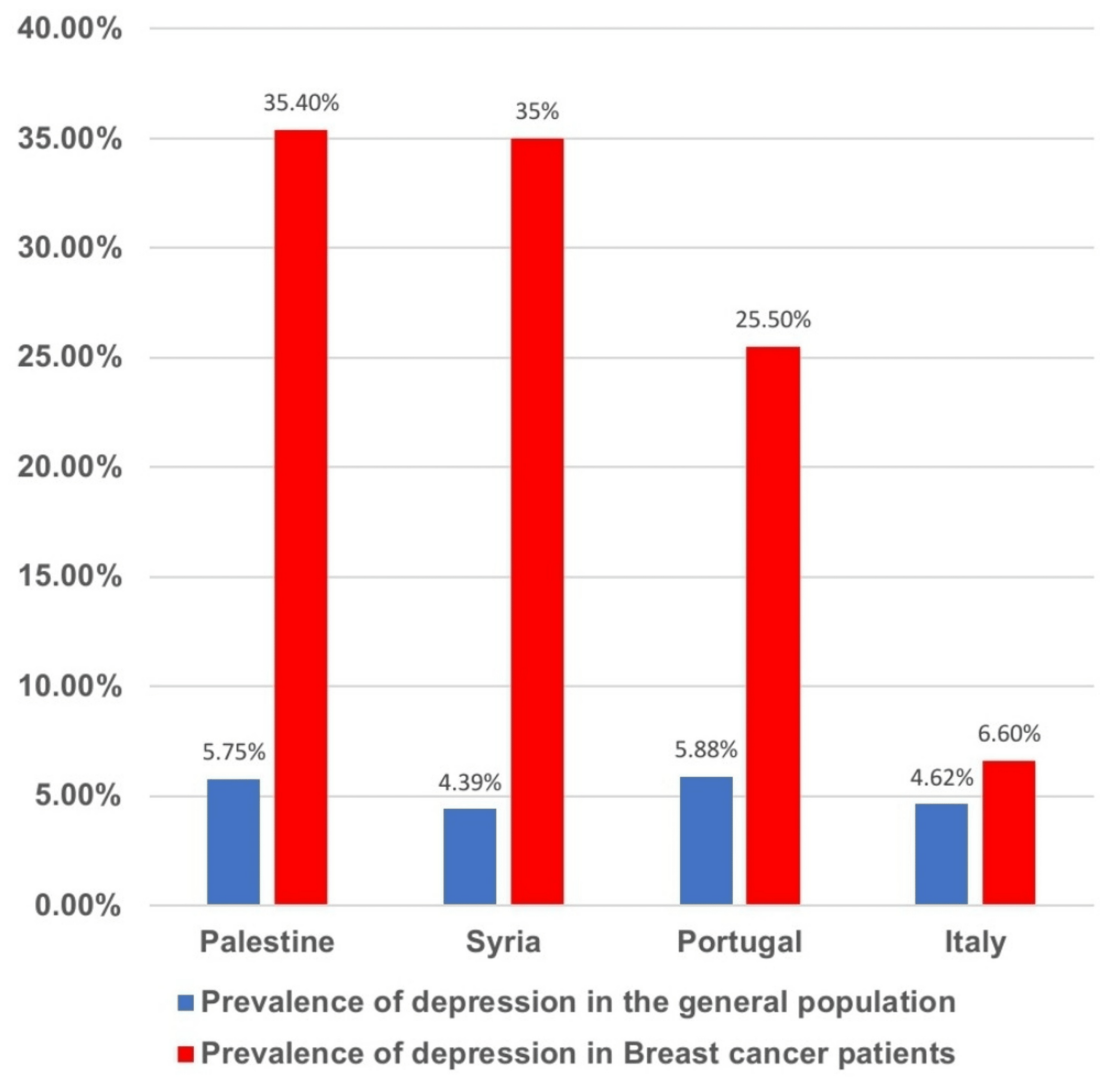
Prevalence of depression among the general population and among the breast cancer patients

**Figure 3:**
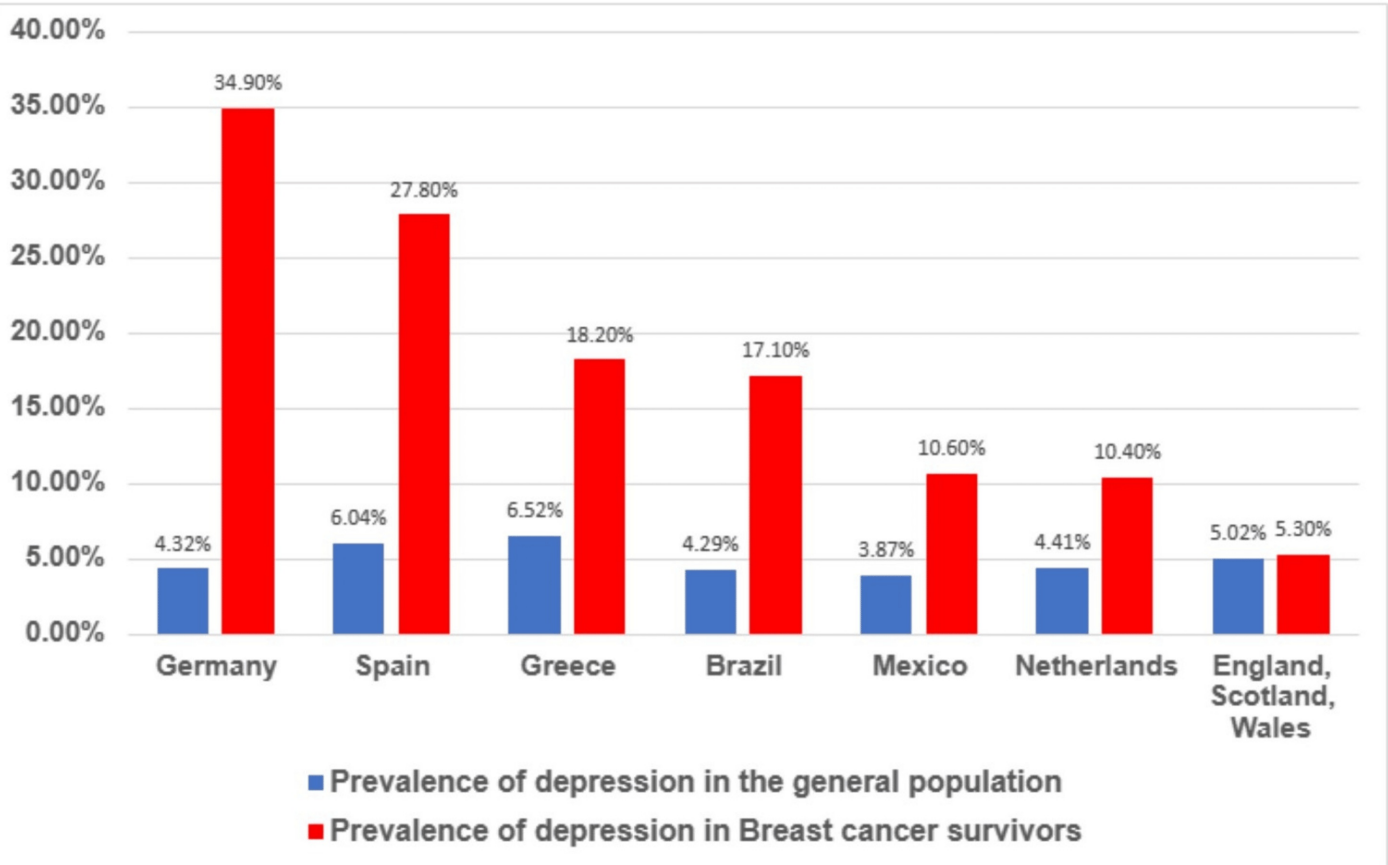
Prevalence of depression among the general population and the breast cancer survivors

Prevalence rates of depression in breast cancer patients in our studies are 6.6% in Italy, 25.5% in Portugal, 35.0% in Syria, and 35.4% in Palestine [7,11,15,16]. Among the studies done on patients, Italy shows the lowest prevalence rates, followed by Portugal, and then Syria and Palestine had nearly the same as well as the highest prevalence rates of depression. The studies that recorded these prevalence rates among breast cancer survivors reported 5.3% in England, Scotland, and Wales, 10.4% in the Netherlands, 10.6% in Mexico, 17.1% in Brazil, 18.2% in Greece, 27.8% in Spain, and 34.9% in Germany [6,8-10,12-14]. The prevalence shows a gradual increase with England, Scotland, and Wales having the lowest prevalence and Germany having the highest prevalence. These depression prevalence rates among breast cancer patients and survivors are much higher than the ones among the general population in these regions [19]. The prevalence rates of depression among breast cancer survivors are generally lower than those among the patients. Figure 2 and Figure 3 graphically illustrate the depression prevalence rates in these regions among breast cancer patients and survivors and those in the general population.

Factors Influencing Depression Prevalence

Several factors may contribute to the variation of depression among breast cancer patients and survivors. The patients who are actively fighting the disease may be subjected to more stress as they are taking treatments such as chemotherapy, radiotherapy, and/or total or partial mastectomy. According to a study by Sadaqa and companions, 51.4% of the women who learned the stage and type of their cancer were more likely to experience extremely high levels of depression, making it a significant risk factor for these patients [15]. Additionally, it was discovered that these patients dreaded both the cancer and the chemotherapy-based treatment and how both might affect their quality of life. Because 45.5% of the patients experienced many side effects from chemotherapy at once, they were substantially more likely to experience moderate to severe depression. The main side effects, which had a significant negative impact on the patients' quality of life, were alopecia, nausea, vomiting, discomfort, and weariness [15]. It was also seen that the patients who had undergone partial or total mastectomy were more at risk of developing depression compared to the ones who did not [6,15,16].

The impact of the stage of breast cancer on the development of depression was described by a study done by Alagizy and companions that showed that among their 64 patients, 31.8% of early disease patients (stages I and II) had moderate to severe depression whereas 68.2% of advanced disease patients (stages III and IV) had moderate to severe depression [20]. This study concluded that depression may occur during any stage of the illness, indicating the importance of addressing mental health concerns throughout the course of breast cancer treatment and management [20].

In the study done by Jameel Soqia and companions, it was found that 35% of breast cancer patients had depression, indicating the necessity to test them for major depressive disorder. They also highlighted that such high levels might be the result of a variety of factors working together, such as the nation's persistent economic woes, the loss of a loved one, and personal hardships [7].

In a study done by Barbara Muzzatti and companions, it was discovered that in patients, mental health and quality of life were poor at the time they were admitted to the hospital, which later with time improved and was comparable to the normal population when they were again assessed after one year [11]. This is summarized in Table 3 and Table 4 [11].

**Table 3:** Physical and mental comprehensive functioning (N=106) during a hospital stay for breast surgery and one year later: means and standard deviations ^+^p<0.01; comparison with norms ^#^p<0.001 comparisons with norms [11]

	Hospital stay	1-year later	P	Norms [16]
Physical functioning	51.8^+^ (9.5)	47.1 (9.0)	0	48.7
Mental functioning	38.2^#^ (11.0)	45.5 (11.0)	0	44.8
p	0	0.218	-	-

**Table 4:** QoL domains (N=106) during the hospital stay for breast surgery and one year later: means and standard deviations ^a^Values from the comparisons between T0 and t1 ^b^1032 Italian females from the general population [11]

	Hospital stay	1-year later	P^a^	Norms^b^ [16]
Physical functioning	81.3 (25.4)	84.3 (15.2)	0.216	81.02
Role-physical limitation	69.8 (38.7)	58.3 (41.8)	0.028	74.13
Bodily pain	76.8 (23.1)	68.6 (254)	0.008	69.01
General health	65.4 (20.5)	63.3 (21.3)	0.312	63.19
Vitality	58.5 (17.7)	56.2 (19.2)	0.278	57.98
Social functioning	59.6 (25.1)	70.6 (23.2)	0	73.89
Role-emotional limitation	48.7 (38.5)	68.2 (38.6)	0	71.78
Mental health	56.0 (19.3)	67.8 (17.3)	0	62.48

This contrasts with cohort study results by Catarina Lopes and Companion, where depression prevalence rates were highest after one year of treatment at 13% [16]. As the years of treatment increase by more than five years, the depression scores have shown to decrease (5.94 at less than one year of treatment vs 3.00 at more than five years of treatment), because the patients start considering it a normal part of life and learn to live with it [10]. 

Age at the time of diagnosis is a significant determinant of the development of depression. It has been found that younger patients are more prone to develop depression compared to the older population [7,8,10,14]. This may be due to younger women having more responsibilities in life such as being the bread earners of their families, raising their children, and having families that depend on them both emotionally and economically [8,10]. Younger people are also found to be more vulnerable because they are very conscious of their physical image and treatments such as breast removal can impact the perceived standards of attractiveness [6]. One study has demonstrated this by correlating the levels of monoamines such as serotonin and dopamine with the age of the patients [14]. It was found in that study that younger patients were more likely to develop mood disorders due to low levels of dopamine and serotonin [14]. In the study by Sadaqa and Companion, age was not found to be a significant factor but that was because most of their study population consisted of older women so the power of the results among younger patients was decreased [15].

Although one shortlisted study suggested that level of education is not an important factor in the development of depression [7], two more studies have found that women with higher levels of education were less vulnerable to developing depression [7,8,16]. This is because not being able to understand and learn about their disease increases the level of stress and uncertainty among women of lower educational backgrounds [8,16]. Another related factor for the development of depression is the employment status of the patients and the survivors [6,10,16]. Women who earn a living cannot work well while they are sick or constantly worry about the recurrence of the disease once the cancer is gone. This increases the financial burden added to the cost of paying for the expensive treatments that they must undergo [10,16].

In a study by Murray Foster and Claire L. Niedzwiedz, it was demonstrated that women with comorbidities are more likely to develop depressive symptoms compared to the ones without, and as the number of comorbidities increased the chances of developing major depressive disorder increased [12]. The highest depression rates were found among patients with comorbid irritable bowel syndrome (11.9%), diabetes (11.6%), and migraine (10.1%) [12]. Similar findings were found in other shortlisted studies by Aggeli and companions and by Breidenbach and colleagues [8,10].

Methodological Considerations

The studies that were included accounted for most of the confounders so the internal validity of these studies is ensured [6-16]. The questionnaires used by these studies were of good quality and the assessment tools for measuring the depression levels were standardized and tested multiple times [6-16]. The sample sizes of the included populations were appropriate in most of the studies and were ethically approved by appropriate approval agencies. The studies by AA Lemij as well as by Catarina Lopes are cohort studies that allow the measurement of a causal relationship between the variables under study [9,16]. The cohort of the study by Catarina Lopes had a 92.1% retention rate, which contributed to the validity of the results [16].

Although the studies that were chosen for our systematic review were very carefully screened, they still have certain limitations. Five of the studies were carried out using a study population from only one healthcare facility, so the results cannot be generalized to the general population of that country or the world [7,10,11,14,16]. The studies included that were based on cross-sectional design cannot establish the causal relationship between depression and breast cancer [6-8,10-15]. The cross-sectional study done by Sergio Álvarez-Pardo on Mexican women cannot be applied to women who have received psychological treatment because these were not included due to unavailability in the study population [6]. In the study by Jameel Soqia, no data was collected on the cancer staging and the treatments given to the patients, which could be the potential confounders in the study [7].

Implications for Clinical Practice

Keeping in mind the high prevalence rates among breast cancer patients and survivors, it is of utmost importance that these people should be routinely screened for depression. Regular assessments using validated depression screening tools can help identify individuals at risk and facilitate early intervention.

Oncologists should be well-trained to look for signs of depression in these patients and they should work closely with psychologists and psychiatrists to provide timely referrals and interventions. Healthcare professionals should educate patients about the potential emotional challenges they might face throughout their breast cancer journey. Educating them about the signs of depression and the importance of seeking help can reduce stigma and encourage early intervention.

Hospitals and healthcare facilities should establish and promote psychological support programs for these patients where they are provided individual sections as well as group therapies, support groups, and counseling sections. It should be recognized that every patient is different and has different risk factors for depression such as age, type of treatment, level of education, employment status, and geographical distribution. So, the therapy approach should be planned accordingly.

Strengths and Limitations of the Systematic Review

Regarding the strengths of our systematic review, it should be noted that it was done according to the PRISMA checklist; the studies we included were good quality studies and included after the quality appraisal was done using the Newcastle-Ottawa scale. We utilized research from 2020 to 2023 so that the data we collected is new and updated. We were able to review the depression prevalence rates in many geographical locations and highlight the important factors that influenced these rates among breast cancer patients and survivors. One of the limitations of our systematic review is that we have not included articles that included the male patients and survivors so our findings cannot be applied to the male population. The other limitation is that we included five articles in which the data was collected from only one healthcare facility; there is a risk that the findings may be influenced by factors unique to that facility, such as its location, patient demographics, or healthcare practices. Therefore, the ability to generalize the results to a wider population, or even to the entire population of that particular country or the world, may be limited. But this has been compensated for by including studies from 13 different countries. By incorporating data from diverse locations, the aim is to enhance the external validity of our findings. Including a variety of countries helps account for regional variations, cultural differences, and other factors that might influence the relationship being studied. This broader scope increases the likelihood that the results can be generalized to a more diverse and representative population.

Recommendations for Future Research

Regarding future research, it is recommended that when measuring the prevalence rates of depression among breast cancer patients and survivors in an area, data should be collected not only from one source but rather from three or more hospitals or healthcare facilities to enhance the quality, validity, and generalizability of research findings. It is also suggested that more research is done on finding out the depression rates among male breast cancer patients and survivors.

## Conclusions

We conducted this systematic review to find out the frequency of depression in the patients and survivors of breast cancer. Through our extensive scrutiny, we were able to conclude that depression prevalence rates were high in the patients as well as the survivors of this cancer. We also found out that higher levels of depression were associated with younger age, positive employment history, lower education, history of chemotherapy, and history of mastectomy in women. Depression can start at any stage of breast cancer and is often overlooked during the discussion of the consequences of breast cancer with the patients as well as with the survivors so they are often blindsided when they begin to experience the symptoms. Through this research paper, we hope to educate healthcare workers about this topic. So, in the future doctors make it a priority to look out for the signs of depression among these individuals and help them with early detection and interventions. We recommend the need for more cohort studies to be done so that more specific data can be collected about the risk factors, preventions, and treatments of depression specifically in patients and survivors of breast cancer.
